# Supplementary material for: Complex‐centric proteome profiling by SEC‐SWATH‐MS
Source: Mol Syst Biol. 2019 Jan 14;15(1):e8438. doi: 10.15252/msb.20188438 (PMC6346213; doi:10.15252/msb.20188438)
Supplement: Supplementary file 8 — Dataset EV7 [file MSB-15-e8438-s008.zip › feature_plots_string/Q9NY93.pdf]

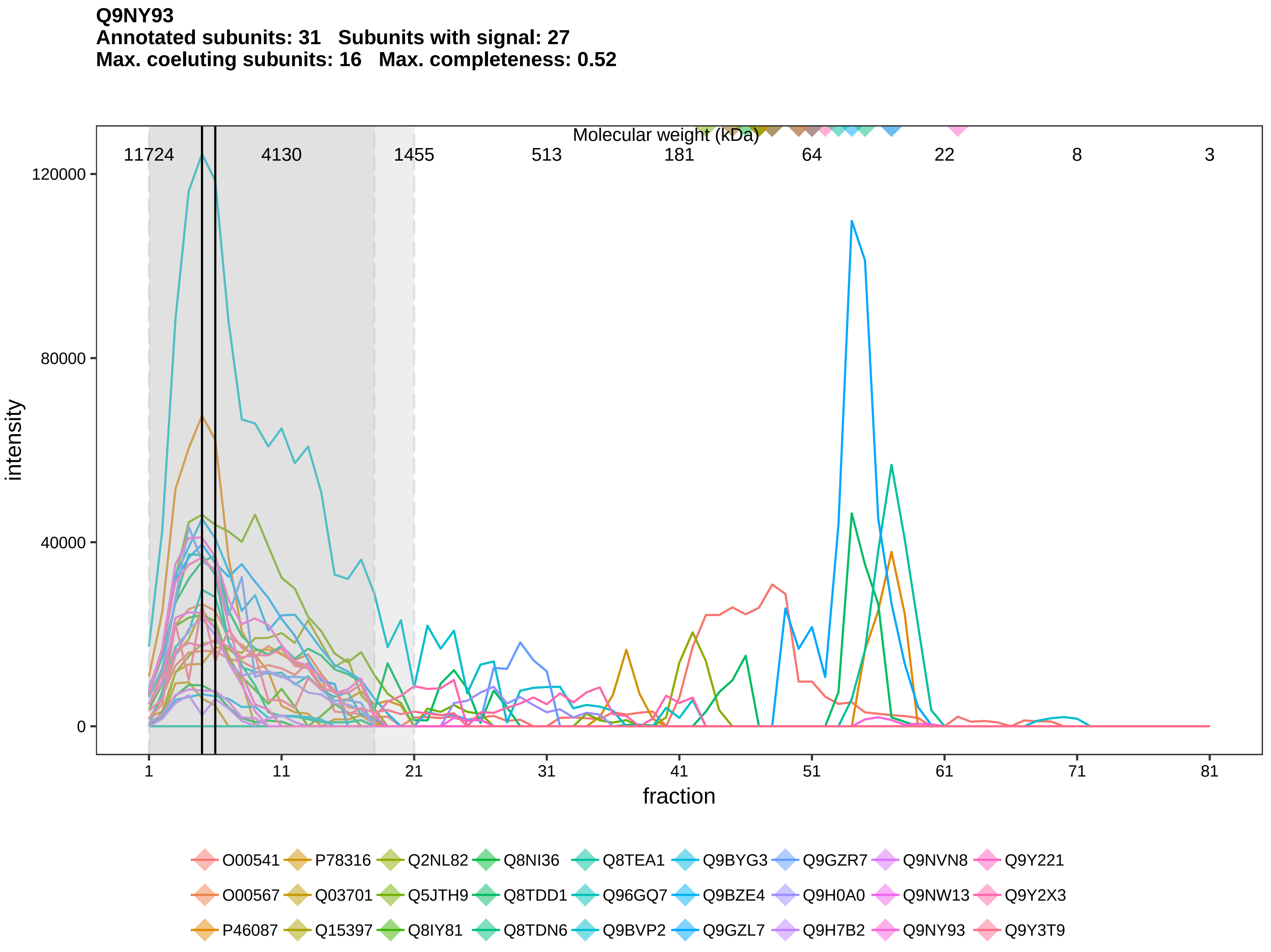

Q9NY93
Annotated subunits: 31 Subunits with signal: 27
Max. coeluting subunits: 16 Max. completeness: 0.52
Molecular weight (kDa)
11724
4130
1455
513
181
64
22
8
3
120000
80000
40000
0
intensity
1
11
21
31
41
51
61
71
81
fraction
O00541
P78316
Q2NL82
Q8NI36
Q8TEA1
Q9BYG3
Q9GZR7
Q9NVN8
Q9Y221
O00567
Q03701
Q5JTH9
Q8TDD1
Q96GQ7
Q9BZE4
Q9H0A0
Q9NW13
Q9Y2X3
P46087
Q15397
Q8IY81
Q8TDN6
Q9BVP2
Q9GZL7
Q9H7B2
Q9NY93
Q9Y3T9
